# DAPK3 participates in the mRNA processing of immediate early genes in chronic lymphocytic leukaemia

**DOI:** 10.1002/1878-0261.12692

**Published:** 2020-05-03

**Authors:** Fraser Thomas, Katie B. Holmes, Sarah Kreuz, Peter Hillmen, Pascal F. Lefevre

**Affiliations:** ^1^ Division of Haematology and Immunology Leeds Institute of Medical Research at St. James's University of Leeds UK

**Keywords:** CLL, DAPK3, H3T11, histone phosphorylation, Ibrutinib, mRNA processing

## Abstract

Cross‐linking of the B‐cell receptor (BCR) induces transcriptional activation of immediate early genes (IEGs) including *EGR1* and *DUSP2* in chronic lymphocytic leukaemia (CLL). Here, we have shown that this transcriptional activation correlated with histone H3 threonine 6 and 11 phosphorylation. Both transcription and histone post‐translational modifications are repressed by ibrutinib, a small molecule inhibitor used in CLL treatment. Moreover, we have identified the death‐associated protein kinase 3 (DAPK3), as the kinase mediating these histone phosphorylation marks in response to activation of the BCR signalling pathway with this kinase being recruited to RNA polymerase II in an anti‐IgM‐dependent manner. DAPK inhibition mimics ibrutinib‐induced repression of both IEG mRNA and histone H3 phosphorylation and has anti‐proliferative effect comparable to ibrutinib in CLL *in vitro*. DAPK inhibitor does not repress transcription itself but impacts on mRNA processing and has a broader anti‐tumour effect than ibrutinib, by repressing both anti‐IgM‐ and CD40L‐dependent activation.

## Graphical abstract

Transcription involves alteration of histones, the proteins surrounding DNA. Here, we show that phosphorylation of histone H3 mediated by the protein DAPK3 does not impact on transcription itself, but is required for co‐transcriptional mRNA processing of key pro‐proliferative genes. Inactivation of DAPK3 not only prevents mRNA processing of these genes but correlates with accumulation of their unprocessed pre‐mRNA transcripts.

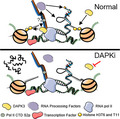

**Abbreviations**
(U/M)‐CLL: (unmutated/mutated)‐chronic lymphocytic leukaemia
BCR: B‐cell receptor
BTK: Bruton's tyrosine kinase
Co‐IP: co‐immunoprecipitation
CpG ODN: CpG oligodeoxynucleotides
DAPK1: death‐associated protein kinase 1
DAPK3: death‐associated protein kinase 3
DAPKi: DAPK inhibitor
DLBCL: diffuse large B‐cell lymphoma
DUSP2: dual‐specificity phosphatase 2
EGR1: early growth response 1
GAIT: interferon‐gamma‐activated inhibitor of translation
GAPDH: glyceraldehyde 3‐phosphate dehydrogenase
H3T11‐P: histone H3 threonine 11 phosphorylation
H3T6‐P: histone H3 threonine 6 phosphorylation
IEG: immediate early gene
IFNγ: interferon‐gamma
IgHV: immunoglobulin heavy chain variable (region)
Kb: kilobase
MYD88: myeloid differentiation primary response 88
NF‐κB: nuclear factor‐kappa B
NLC: nurse‐like cell
PLCγ2: phospholipase Cγ2
PPP6C: protein phosphatase 6 catalytic subunit
PTM: post‐translational modification
qPCR: quantitative polymerase chain reaction
RNA pol II S2‐P: RNA polymerase II serine 2 phosphorylation
RPMI: Roswell Park Memorial Institute (media)
siRNA: small interfering ribonucleic acid
TBP: TATA‐box binding protein
TLR: Toll‐like receptor
TSS: transcription start site
ZIPK: zipper‐interacting protein kinase

## 1 Introduction

Bruton's tyrosine kinase (BTK) inhibitor ibrutinib has shown extremely positive results in the treatment of chronic lymphocytic leukaemia (CLL; Burger *et al.*, 2015; Byrd *et al.*, 2014) and is now being tested in combination with other therapies (Collett *et al.*, 2017; Hillmen *et al.*, 2019; Kater *et al.*, 2019). However, cases of resistance are emerging (Ahn *et al.*, 2017; Kaur and Swami, 2017) and the long‐term outcome is generally poor after treatment discontinuation (Jain *et al.*, 2017). Ibrutinib‐mediated relapse in CLL is mostly associated with genomic alterations in *BTK* and its downstream target *PLCγ2*, but cases of progressive disease in which targeted sequencing is unable to explain resistance have also been reported (Landau *et al.*, 2017).

Chronic lymphocytic leukaemia can be divided into two main subsets, depending on whether the leukaemic cells arise from a B cell before somatic hypermutation (U‐CLL) or after (M‐CLL), the latter being generally less aggressive than the former (Damle *et al.*, 1999; Hamblin *et al.*, 1999). The tissue microenvironment, a distinct anatomical proliferation centre in the lymph node, plays an important role in the pathogenesis of CLL (Burger, 2011). Within these centres, CLL cells thrive from the external pro‐survival signals they receive from stromal, nurse‐like cells (NLCs) and T cells leading to unique gene expression profiles with substantial activation of nuclear factor‐kappa B (NF‐κB) and pro‐survival signalling cascades from the B‐cell receptor (BCR) (Burger, 2011; Choi *et al.*, 2016; Herishanu *et al.*, 2011). The importance of the microenvironment is highlighted by the clinical efficacy of ibrutinib which inhibits BCR signalling to disrupt trafficking of CLL cells to the lymphoid organs (Choi *et al.*, 2016). A significant number of cases of both primary and secondary resistance to this treatment have been described (Ahn *et al.*, 2017; Kaur and Swami, 2017). However, activation of alternative signalling pathways to bypass BTK inhibition may also play a role in the development of resistance to ibrutinib. For example, it has been reported that Toll‐like receptor (TLR) signalling in the lymph node is only partially repressed by ibrutinib (Dadashian *et al.*, 2019). Furthermore, combining ibrutinib with the BCL‐2 inhibitor venetoclax is a proven and successful treatment option in CLL (Hillmen *et al.*, 2019; Kater *et al.*, 2019), yet resistance can still arise from strong activation of the NF‐κB pathway via microenvironmental agonists such as CD40L and CpG oligodeoxynucleotides (Jayappa *et al.*, 2017). Similarly, WNT5A, a secreted glycoprotein belonging to the WNT family, can activate RAC1 and lead to proliferation in CLL and mantle cell lymphoma, a process which is not repressed by ibrutinib (Yu *et al.*, 2017, 2018).

Therefore, intensive efforts have been undertaken to better understand the mechanism of response to ibrutinib and the development of drug resistance. We have previously reported an important epigenomic plasticity of CLL cells in response to ibrutinib treatment (Holmes *et al.*, 2019). In the present analysis, focussing on two immediate early genes (IEG) stimulated in response to BCR signalling activation with anti‐IgM, we observed that ibrutinib‐dependent transcriptional repression of these genes correlated with a block in histone H3 threonine 6 and 11 (H3T6 and H3T11) phosphorylation. We identified death‐associated protein kinase 3 (DAPK3), also known as zipper‐interacting protein kinase (ZIPK), as the enzyme mediating these histone H3 post‐translational modifications (PTMs). DAPK inhibition (DAPKi) impairs mRNA processing and CLL cell proliferation. Finally, in contrast to ibrutinib, DAPKi prevents transcriptional activation induced by both anti‐IgM and CD40L suggesting that targeting this kinase might have a wider effect than ibrutinib. In conclusion, targeting DAPK3 might be a suitable alternative to BTK inhibitors in the treatment of CLL, including in patients carrying *BTK* or *PLCγ2* mutations.

## 2 Methods

### 2.1 Cell culture and siRNA knockdown

Chronic lymphocytic leukaemia cells were obtained from the St James's University Hospital (Leeds) Haematological Malignancy Diagnostic Service (HMDS) from patients with no previous treatment for their disease. The experiments using these cells were undertaken with the understanding and written consent of each patient and the study methodologies conformed to the standards set by the Declaration of Helsinki. These experiments were performed under ethical approval granted by the Leeds Teaching Hospital NHS Trust REC: 14/WS/0098.

Chronic lymphocytic leukaemia and HBL1 (DLBCL cell line) cells were cultured in Roswell Park Memorial Institute (RPMI‐1640; Sigma, St. Louis, MO, USA) medium with 10% fetal bovine serum (PAA Laboratories Inc., Toronto, ON, Canada), l‐glutamine (Thermo Fisher; Gibco™, Dublin, Ireland) and penicillin‐streptomycin (Thermo Fisher; Gibco™). CLL peripheral blood mononuclear cells were isolated by density centrifugation from whole blood using Lymphoprep™ (Stemcell Technologies, Vancouver, Canada). CLL cells were cultured on a layer of CD40L‐expressing feeder cells where indicated. Cells were stimulated with anti‐IgM at 10 μg·mL^−1^ (Jackson‐ImmunoResearch, West Grove, PA, USA; 109‐006‐129‐JR) or recombinant human sCD40 ligand (PeproTech, London, UK; 310‐02) at 5 μg·mL^−1^ as required and where indicated. Cells were pretreated with ibrutinib (Pharmacyclics, Sunnyvale, CA, USA) at 1 μm or a DAPK inhibitor (DAPKi) (Calbiochem, San Diego, CA, USA; 324788‐10MG) at 10–120 μm as required and where indicated. DAPK3 knockdown was achieved in HBL1 cells with a GenePulser^®^ II electroporation system (Bio‐Rad, Hercules, CA, USA) using siRNAs against DAPK3 (Thermo Fisher, Waltham, MA, USA; siRNA ID #557 and #559) complete with a nontargeting negative control siRNA (Thermo Fisher; 4390843). siRNA transfected cells were incubated for 3–5 days with fresh RPMI on day 1 and 3. For the cell survival assay, cells were stained with trypan blue (Thermo Fisher) and counted using a haemocytometer on the indicated day post‐seeding.

### 2.2 cDNA preparation, qPCR and RT‐PCR

Total RNA was prepared using TRIzol^®^ reagent (Invitrogen, Carlsbad, CA, USA) according to the manufacturer's recommendations. RNA was prepared with Direct‐zol™ RNA MiniPrep kit (Zymo, Irvine, CA, USA). cDNA was synthesised with Random Primers (Invitrogen) or Oligo(dT) (Invitrogen), 5× FS buffer (Invitrogen), MLV‐reverse transcriptase (Invitrogen), RNase‐Out (Invitrogen) and dNTPs (Invitrogen). qPCR reactions were carried out using Luna^®^ Universal qPCR Master Mix (NEB, Ipswich, MA, USA) on a QuantStudio 7 Flex Real‐Time PCR System (Thermo Fisher). Relative expression was calculated as a ratio of specific transcript to one/several housekeeping genes: TATA‐box binding protein (*TPB*), protein phosphatase 6 catalytic subunit (*PPP6C*) or glyceraldehyde 3‐phosphate dehydrogenase (*GAPDH*), as indicated. RT‐PCR reactions were carried out using a thermal cycler (Bio‐Rad). RT‐PCR products were run on 1.5% agarose gels for 30–40 min at 100 V and visualised with a ChemiDoc system. See Table S1 for primer sequences.

### 2.3 ChIP and qPCR analysis

ChIP was performed as follows: 10^7^ cells were cross‐linked with 1.5% formaldehyde for 8 min at room temperature. Cells were harvested by centrifugation at 400 ***g*** for 4 min at 4 °C and washed twice with ice cold PBS supplemented with 1× protease inhibitor cocktail (NEB; 5871S). Pellets were resuspended in 10 mL of buffer A [10 mm HEPES (pH 8), 10 mm EDTA (pH 8.0), 0.5 mm EGTA (pH 8.0) and 0.25% Triton X‐100] and incubated at 4 °C for 10 min with gentle agitation. After centrifugation at 500 ***g*** at 4 °C for 5 min, cells were resuspended into 40 mL of buffer B [10 mm HEPES (pH 8), 200 mm NaC1, 1 mm EDTA (pH 8.0), 0.5 mm EGTA (pH 8.0) and 0.01% Triton X‐100] and incubated 10 min, and centrifuged as before. Nuclei were sonicated in immunoprecipitation buffer [25 mm Tris/HCl (pH 8), 2 mm EDTA, 150 mm NaC1, 1% Triton X‐100, 0.1% sodium dodecyl sulfate and 1× protease inhibitor cocktail]. Nuclei were sonicated for 15 (RNA polymerase ChIP) or 20 (histone H3 ChIP) cycles (30 s on/30 s off) using a Bioruptor^®^ Pico sonication device (Diagenode, Liège, Belgium). After centrifugation at 14 000 ***g*** for 10 min at 4 °C, chromatin preparations were stored at –80 °C. Twelve microlitre of chromatin was taken and stored as input for qPCR normalisation.

Sonicated chromatin from 10^7^ cells was used for each immunoprecipitation. The volume was adjusted to give 100 μL per IP with fresh immunoprecipitation buffer. Immunoprecipitation was achieved using Dynabeads™ Protein G (Thermo Fisher) with 2.4 µg of anti‐histone H3T11‐P (Abcam, Cambridge, UK; ab5168), anti‐histone H3T6‐P (Diagenode; C15410282), anti‐RNA polymerase II (Abcam; ab817) or anti‐RNA polymerase II S2‐P (Abcam; ab5095) per 10 µL of beads. Beads were washed twice with ice cold immunoprecipitation buffer prior to use. Beads and antibodies were incubated for 2 h at 4 °C with gentle agitation. After 2 h, the bead–antibody mixture was pelleted using a magnet and the beads were washed once with ice cold immunoprecipitation buffer. About 100 µL of sonicated chromatin was added to achieve a bead to chromatin ratio of 1 : 10. Chromatin was incubated for 2 h at 4 °C with gentle agitation. After 2 h, beads were pelleted using a magnet and washed once with washing buffer A [20 mm Tris/HCl (pH 8), 2 mm EDTA, 1% Triton X‐100, 0.1% sodium dodecyl sulfate and 150 mm NaCl supplemented with 1× protease inhibitor cocktail), once with washing buffer B (20 mm Tris/HCl (pH 8), 2 mm EDTA, 1% Triton X‐100, 0.1% sodium dodecyl sulfate and 500 mm NaCl supplemented with 1× protease inhibitor cocktail], once with LiCl buffer [0.25 m LiCl, 0.5% NP‐40, 0.5% sodium deoxycholate, 1 mm EDTA and 10 mm Tris/HCl (pH 8) supplemented with 1× protease inhibitor cocktail], and twice with TE buffer [10 mm Tris/HCl (pH 8.0) and 1 mm EDTA]. The immune complexes were eluted by adding 50 μL of elution buffer (1% sodium dodecyl sulfate, 100 mm NaHCO_3_). The cross‐link was reversed at 65 °C overnight. Inputs were treated with RNAse A for 30 min at 37 °C and reverse cross‐linked at 65 °C overnight with proteinase K.

Reverse cross‐linked DNA was purified using AxyPrep™ MAG PCR clean‐up kit (Appleton Woods, Birmingham, UK) according to manufacturer's instructions. qPCR reactions were carried out using Luna^®^ Universal qPCR Master Mix (NEB) on a QuantStudio 7 Flex Real‐Time PCR System (Thermo Fisher). See Table S1 for primer sequences.

### 2.4 Co‐immunoprecipitation

For co‐immunoprecipitation (co‐IP), cells were lysed with cold immunoprecipitation buffer (IP buffer) (150 mm NaCl, 10 mm Tris/HCl pH 7.4, 1 mm EDTA, 1 mm EGTA, 1% Triton X‐100 and 0.5% NP‐40) complete with 1× protease inhibitor cocktail (NEB; 5871S) for 30 min at 4 °C maintaining agitation. Lysates were centrifuged, and supernatants were quantified using the BCA Protein Assay (Pierce™, Waltham, MA, USA) according to manufacturer's instructions and a Mithras Plate Reader (Berthold Technologies, Bad Wildbad, Germany). Cell lysates were adjusted to 1 mg·mL^−1^ with IP buffer. Lysates were precleared with Dynabeads™ Protein G (Thermo Fisher) for 1 h or overnight at 4 °C while maintaining agitation. Supernatants were incubated with 1–5 μg of anti‐histone H3 (Abcam; ab1791), anti‐RNA polymerase II (Abcam; ab817), anti‐RNA polymerase II S2‐P (Abcam; ab5095) or HRP‐conjugated anti‐Rat IgG (eBioscience, San Diego, CA, USA; 18‐4818‐12) for 1 h at 4 °C maintaining agitation. The antibody‐lysate immune complexes were captured with 12.5 μL Dynabeads™ Protein G (Thermo Fisher) per 100 μg lysate by maintaining agitation for 1 h at 4 °C. Immobilised complexes were washed up to five times with IP buffer and eluted in 4× SDS/PAGE loading buffer (200 mm Tris/HCl pH 6.8, 400 mm DTT, 8% SDS, 0.4% bromophenol blue and 40% glycerol) at 70 °C for 5 min. Samples were electrophoresed on SDS/polyacrylamide gels (PAGE) and detected with primary antibodies by western blot (see below).

### 2.5 Western blot

For whole cell lysate preparation, cells were washed twice with ice cold PBS, centrifuging at 124 ***g*** for 5 min. Samples were lysed with SDS lysis buffer (10% SDS, 0.5 m Tris pH 6.8) supplemented with 1× protease inhibitor cocktail (NEB; 5871S), PMSF (1 µL·mL^−1^), NaF (1 µL·mL^−1^) and Na_3_VO_4_ (2 µL·mL^−1^) for 5 min at 90 °C. Protein concentrations of all samples were determined using the BCA Protein Assay (Pierce™) according to manufacturer's instructions and a Mithras Plate Reader (Berthold Technologies). Western blots were carried out under denaturing conditions with SDS/PAGE, and proteins were wet‐transferred to nitrocellulose membranes. Membranes were blocked with either 5% BSA or 5% milk for 1 h at room temperature. Membranes were incubated at 4 °C overnight with primary antibodies against DAPK3 at 1 : 1000 (Bethyl; A304‐222A‐1), DAPK3‐T265P at 1 : 500 (Abcam; ab63395), anti‐histone H3T11‐P at 1 : 500 (Abcam; ab5168), anti‐histone H3T6‐P at 1 : 500 (Diagenode; C15410282), anti‐EGR1 (CST, Danvers, MA, USA; #4153) at 1 : 500 or anti‐β‐actin at 1 : 2000 (Thermo Fisher; PA1‐183). HRP‐conjugated secondary antibodies against rabbit (Jackson‐ImmunoResearch; 111‐035‐003) and mouse (Jackson‐ImmunoResearch; 315‐035‐003) were used at 1 : 10 000. Membranes were analysed using ECL (Thermo Fisher; Pierce™) and a ChemiDoc system. Bands were quantified with image lab™ software (BioRad) as indicated.

### 2.6 Kinase assay

0.1 μg recombinant protein DAPK3‐GST (Abcam; ab152327) and 1 μg recombinant protein histone H3‐HIS (Abcam; ab198757) were added to kinase buffer (KB) (1 mg·mL^−1^ BSA, 25 mm HEPES pH 7.9, 10 mm MgCl_2_, 50 mm NaCl, 1 mm DTT, 10 mm KCl and 10% glycerol) complete with 1× protease inhibitor cocktail (NEB; 5871S). ATP was added to 200 μm final concentration. Ibrutinib was added to 1 μm final concentration. DAPKi was added to 25 μm final concentration. Samples were warmed to 16 °C to start the reaction. Aliquots were removed at the indicated time points and immediately heat inactivated for 5 min at 65 °C to halt the reaction. Reaction samples were spotted onto nitrocellulose membranes and air dried before blocking in 5% BSA at room temperature for 1 h. Membranes were incubated at 4 °C overnight with anti‐histone H3T11‐P (Abcam; ab5168) and anti‐histone H3T6‐P (Diagenode; C15410282). HRP‐conjugated secondary antibodies against rabbit (Jackson‐ImmunoResearch; 111‐035‐003) and mouse (Jackson‐ImmunoResearch; 315‐035‐003) were used at 1 : 10 000. Membranes were analysed using ECL (Thermo Fisher; Pierce™) and a ChemiDoc system. Dots were quantified with image lab™ software (BioRad) as indicated.

### 2.7 Flow cytometric analysis

Cells were analysed on a 3 laser BD LSR II flow cytometer (BD Biosciences, Franklin Lakes, NJ, USA). CLL cell apoptosis analysis was carried out using the FITC Annexin V Apoptosis Detection Kit with 7‐AAD (BioLegend, San Diego, CA, USA) according to the manufacturer's instructions. CLL cell proliferation assays utilised the antibody FITC Mouse Anti‐human Ki‐67 (BD Pharmingen™, Franklin Lakes, NJ, USA; 556026 clone B56). CLL cells were cultured on a CD40L‐expressing feeder layer prior to analysis. Analysis was performed with bd facsdiva Software 5.0 (BD Biosciences) and flowjo (Ashland, OR, USA).

### 2.8 Primers for qPCR, RT‐PCR and ChIP‐qPCR

Primers were designed online using primer3plus (University of Heidelberg, Germany) and the NCBI primer‐blast (Bethesda, MD, USA) tool in conjunction with the *in silico* PCR function of the UCSC genome browser (Table S1). The number following a primer indicates the distance from the transcription start site (TSS) in kilobases (kb) of the corresponding gene where negative values are upstream of the TSS and positive values are downstream of the TSS.

## 3 Results

### 3.1 Phosphorylation of histone H3 is induced by BCR cross‐linking at IEGs loci

**Fig. 1:**
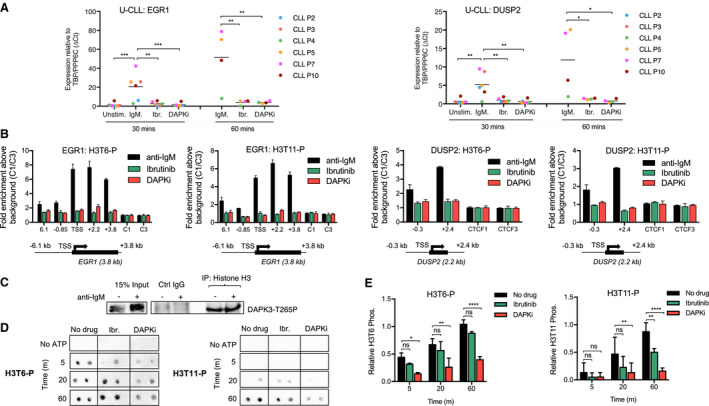
H3T6 and H3T11 phosphorylation at IEGs is catalysed by DAPK3 and is similarly abrogated by both BTK and DAPK3 inhibition. (A) qPCR data analysis of EGR1 and DUSP2 gene expression at 30 (*n* = 6 patients) and 60 min (*n* = 4 patients) post‐anti‐IgM stimulation in U‐CLL cells. CLL cells were pretreated with 1 µm ibrutinib or 25 µm DAPKi for 1 h as indicated below the graphs before anti‐IgM stimulation. Expression changes were quantified using the ∆*C*_t_ method with TBP and PPP6C as control genes. Bars represent the grand mean of the samples. Significant differences calculated using two‐way ANOVA followed by Dunnett's multiple comparison test with anti‐IgM as control. EGR1 (30 m) *P* values = 0.0009, 0.0015 and 0.0009 for anti‐IgM vs Unstim, Ibr and DAPKi respectively. DUSP2 (30 m) *P* values = 0.0013, 0.0023 and 0.0011 for the same comparisons. EGR1 (60 m) *P* values = 0.0039, 0.0052 and 0.0052 for the same comparisons. DUSP2 (60 m) *P* values = 0.0204, 0.0269 and 0.0205 for the same comparisons. (B) ChIP‐qPCR data assessing levels of H3T6‐P and H3T11‐P at the EGR1 and DUSP2 gene loci. CLL cells pretreated with either 1 µm ibrutinib or 25 µm DAPKi for 1 h before stimulation with anti‐IgM for 30 min. Values on the *x* axis refer to specific gene regions relative to the TSS in kilobases (kb) as indicated on the gene schematics below (not to scale). Error bars represent the SD of three independent experiments. CTCF1/3 (C1/C3) were used as negative control regions which are not indicated on the gene schematics. (C) co‐IP of CLL cells stimulated with anti‐IgM for 1 h as indicated. Immunoprecipitates from histone H3 pulldown were analysed by SDS/PAGE followed by western blot probing for DAPK3‐T265P. Untreated, crude cell lysate was used as positive control (input), and IgG beads were used for negative control (Ctrl IgG). Blot is representative of three independent co‐IP experiments. Western blot is cropped due to different exposure times for input and co‐IP lysates. (D) Kinase assay quantified by dot‐blot with recombinant histone H3 and recombinant DAPK3 to measure DAPK3 kinase activity over time (5–60 min) with or without (negative control) ATP and in the presence of 1 µm ibrutinib and 25 µm DAPK inhibitor. (E) Kinase assay displaying histone H3 phosphorylation quantified relative to untreated assay with error bars representing the SD of three independent experiments. Significant differences calculated using two‐way ANOVA followed by Dunnett's multiple comparison test with the untreated sample as control for each time point. H3T6‐P *P* values = 0.3075 and 0.0139 (5 m), 0.4157 and 0.0021 (20 m), 0.1583 and 0.0001 (60 m) for untreated vs. Ibr and DAPKi, respectively. H3T11‐P *P* values = 0.6679 and 0.6844 (5 m), 0.0672 and 0.0095 (20 m), 0.0043 and 0.0001 (60 m) for the same comparisons. Ns *P* > 0.05, * *P* ≤ 0.05, ** *P* ≤ 0.01, *** *P* ≤ 0.001.

The link between signalling‐dependent chromatin alterations and changes in gene expression is poorly understood. To better understand, how chromatin structure responds to BCR cross‐linking, we performed a screening of histone H3 phosphorylation marks induced after anti‐IgM stimulation in primary CLL cells and identified two phospho‐marks (H3T6p and H3T11p) enriched at IEGs and correlated with expression of these genes. To further analyse these chromatin structure alterations, we chose two genes as markers of response to anti‐IgM in U‐CLL (*EGR1* and *DUSP2*) and investigated the changes in mRNA level and chromatin structure in response to BCR cross‐linking. These two genes were selected after an initial screening for genes responding to anti‐IgM stimulation in primary CLL, and for which the response was repressed by addition of ibrutinib looking at *EGR1, DUSP2, MYC, DUSP4, CCND2, BCL2A1, PRDX1, NCL, CDC25B, TLR10, PIM1, CXCR4, CXCR5, CCR6* and *CCR7*. From these genes, only the first 5 were convincingly stimulated and repressed without or with ibrutinib, respectively. However, *EGR1* and *DUSP2* mRNA level was maximum after 30‐min stimulation whereas, for *MYC, DUSP4* and *CCND2*, this peak occurs between 2 and 4 h post‐stimulation. The IEG early growth response 1 (*EGR1*) controls B‐cell proliferation in response to BCR cross‐linking but not after CD40 ligation or TLR4 activation (Gururajan *et al.*, 2008; McMahon and Monroe, 1996) and correlates with survival in CLL (Stratowa *et al.*, 2001). Dual‐specificity phosphatase 2 (*DUSP2*) is another IEG induced by anti‐IgM stimulation in B cells (Grumont *et al.*, 1996). DUSP2 regulates MAPK activity and plays an essential role in cell proliferation and cancer (Wei *et al.*, 2013). Expression of both genes was activated 30–60 min post‐anti‐IgM stimulation (Fig. 1A) and correlated with enriched phosphorylation of histone H3T6 and H3T11 within the body of these genes (Fig. 1B). In addition, both gene expression and histone H3 phosphorylation were impeded by ibrutinib (Fig. 1A,B). Consequently, this approach represented a reliable and simple method to assessing the cellular response to BCR cross‐linking and ibrutinib‐mediated repression of this signal.

To determine which kinase downstream of the BCR signalling pathway might mediate histone H3 phosphorylation at the indicated threonine residues, we used the *in silico* kinase prediction software GPS (Xue *et al.*, 2008) (Fig. S1A). Among the identified kinases, MEK2, RSK1/2 and PKCs are known downstream elements of the BCR signalling pathway. Consequently, inhibitors targeting these kinases were tested for their ability to inhibit expression of *EGR1* and *DUSP2* and phosphorylation of H3T6 and H3T11 along these genes. The inhibitors used were Gö6983, a pan‐PKC inhibitor, RSK inhibitor II, a pan‐RSK inhibitor, and U0126, a MEK1/2 inhibitor. None of the three inhibitors tested significantly inhibited the induction of *EGR1* or *DUSP2* expression or prevented histone H3 phosphorylation after 30‐ and 90‐min anti‐IgM stimulation (Fig. S2). In contrast, the death‐associated protein kinase inhibitor (DAPKi) was the only compound which showed repression of both anti‐IgM stimulation and histone H3 phosphorylation (Fig. 1A,B). DAPKi is an inhibitor specific against DAPK1 and DAPK3, two enzymes of the DAPK family (Fig. S1B). Epigenetic silencing of DAPK1 by promoter methylation is a characteristic of sporadic CLL (Raval *et al.*, 2007), indicating, therefore, that DAPKi was mainly targeting DAPK3 in these cells. This enzyme was originally identified as a chromatin‐associated enzyme (Kögel *et al.*, 1998) mediating histone H3T11 phosphorylation (Preuss *et al.*, 2003). co‐IP experiments demonstrated that DAPK3 and histone H3 were interacting *in vivo* independently of anti‐IgM stimulation (Fig. 1C), and *in vitro* kinase assay confirmed that DAPK3 could phosphorylate histone H3 at T6 and T11, both effects being repressed by DAPKi (Fig. 1D,E). Our data were therefore in line with the original observations identifying DAPK3 as a chromatin‐associated enzyme inducing mitosis‐specific H3T11 phosphorylation (Kögel *et al.*, 1998; Preuss *et al.*, 2003). In contrast, ibrutinib had no significant impact on DAPK3 activity apart from T11 at 60 min (Fig. 1D,E), which may be due to an indirect effect arising from the addition of ibrutinib to the reaction (e.g. changes in stoichiometry). Altogether, these results showed that DAPK3 was activated downstream of BTK in response to BCR cross‐linking.

### 3.2 DAPK inhibition impacts on mRNA processing

**Fig. 2:**
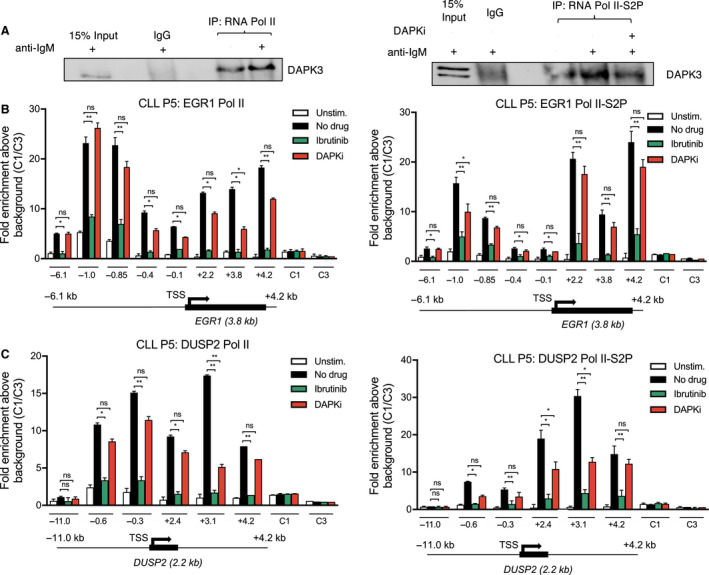
BTK and DAPK3 inhibition have different effects on RNA polymerase II activity at IEGs. (A) co‐IP of HBL1 cells stimulated with anti‐IgM for 1 h as indicated. HBL1 cells were pretreated with 25 µm DAPKi for 1 h where indicated before anti‐IgM stimulation. Immunoprecipitates from RNA polymerase II and RNA polymerase II S2‐P pulldown were analysed by SDS/PAGE followed by western blot probing for DAPK3. Untreated, crude cell lysate was used as positive control (input), and IgG beads were used for negative control (Ctrl IgG). Blots are representative of 3 independent co‐IP experiments. (B, C) ChIP‐qPCR data from CLL cells assessing levels of RNA polymerase II (left) and RNA polymerase II S2‐P (right) binding across the (B) EGR1 and (C) DUSP2 loci at 30 min post‐stimulation with anti‐IgM. CLL cells were pretreated with either 1 µm ibrutinib (green) or 25 µm DAPKi (red) for 1 h as indicated before anti‐IgM stimulation. The values on the *x* axis refer to specific gene regions relative to the TSS in kilobases (kb) as indicated on the gene schematics below (not to scale). CTCF1/3 were used as negative control regions which are not indicated on the gene schematics. Error bars represent standard deviation for three independent ChIP experiments for *n* = 1 CLL patient. Significant differences calculated using two‐way ANOVA followed by Dunnett's multiple comparison test, to compare anti‐IgM with Ibr and DAPKi. Pol II *P* values = 0.0456 (EGR1 −6.1), 0.0056 (EGR1 −1.0), 0.0023 (EGR1 −0.85), 0.0388 (EGR1 −0.4), 0.0498 (EGR1 −0.1), 0.0235 (EGR1 +2.2), 0.0295 and 0.0415 (EGR1 +3.8), 0.0031 (EGR1 +4.2), 0.0318 (DUSP2 −0.6), 0.0081 (DUSP2 −0.3), 0.0410 (DUSP2 +2.4), 0.0037 and 0.0051 (DUSP2 +3.1) and 0.0057 (DUSP2 +4.2). Pol II S2‐P *P* values = 0.0351 (EGR1 −6.1), 0.0097 and 0.0481 (EGR1 −1.0), 0.0036 (EGR1 −0.85), 0.0291 (EGR1 −0.4), 0.0410 (EGR1 −0.1), 0.0046 (EGR1 +2.2), 0.0036 (EGR1 +3.8), 0.0068 (EGR1 +4.2), 0.0398 (DUSP2 −0.6), 0.0086 (DUSP2 −0.3), 0.0261 and 0.0377 (DUSP2 +2.4), 0.0071 and 0.0295 (DUSP2 +3.1) and 0.0055 (DUSP2 +4.2). Graphs are representative of the data obtained from the same experiment in *n* = 3 CLL patients. Ns *P* > 0.05, * *P* ≤ 0.05, ** *P* ≤ 0.01, *** *P* ≤ 0.001.

To further decipher the role of DAPK3 in the transcriptional activation of our two IEGs, we performed co‐IP experiments looking at DAPK3 interaction with total RNA polymerase II (RNA pol II) and the elongating form of the polymerase phosphorylated at serine 2 (RNA pol II S2P) (Fig. 2A). DAPK3 interacted with both forms of the polymerase, and this interaction is enhanced by anti‐IgM stimulation and marginally impaired by addition of DAPKi (Fig. 2A). In addition, looking at RNA pol II recruitment to *EGR1* and *DUSP2* loci in response to anti‐IgM stimulation, we detected an accumulation of total and elongating polymerases at promoters and gene bodies, which is prevented by ibrutinib (Fig. 2B,C). In contrast, no effect was observed after addition of DAPKi, suggesting that transcription elongation was unaffected by this inhibitor.

**Fig. 3:**
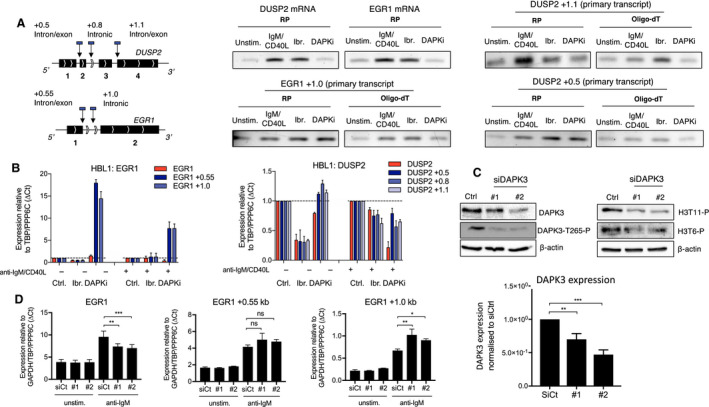
DAPK3‐mediated histone H3 threonine phosphorylation is required for faithful co‐transcriptional splicing. (A) EGR1/DUSP2 gene schematics showing introns and exons. Primer locations for qPCR and RT‐PCR indicated by vertical arrows and blue boxes. Numbers after gene names correspond to the position of the primers in and around the gene relative to the TSS, where + denotes 3′ of the TSS and − denotes 5′ of the TSS. Reverse transcription PCR (RT‐PCR) analysis comparing levels of processed EGR1/DUSP2 mRNA (top left) with EGR1/DUSP2 primary transcript levels (bottom left, top right, bottom right). CLL cells were pretreated with either 1 µm ibrutinib or 25 µm DAPKi for 1 h as indicated and then stimulated with anti‐IgM and sCD40L to activate IEG expression. Extracted RNA was reverse transcribed with either random primer (RP) or oligo‐dT, subjected to RT‐PCR and analysed by agarose gel electrophoresis. (B) qPCR analysis of EGR1/DUSP2 primary transcript (shades of blue) vs processed mRNA (red) from HBL1 cells pretreated with either 1 µm ibrutinib or 25 µm DAPKi for 1 h and then stimulated with or without anti‐IgM and CD40L for 1 h as indicated. Error bars represent the SD of *n* = 3 independent experiments. (C) Western blots of HBL1 cells transfected with siRNAs against DAPK3 (#1 and #2) and with a nonspecific control siRNA (siCtrl). On day 5 post‐transfection, cells were harvested and lysates probed with antibodies against DAPK3, DAPK3‐T265‐P, histone H3T6‐P and histone H3T11‐P. β‐actin was used as a loading control and for normalisation to siCtrl using image lab software (right). Blots are representative of three independent transfections. Significant differences calculated using two‐way ANOVA followed by Dunnett's multiple comparison test. *P* values for DAPK3 expression normalised to siCtrl = 0.0036 and 0.0004 for siCtrl vs. siDAPK3 #1 and siDAPK3 #2, respectively. (D) qPCR analysis of HBL1 cells transfected with siRNAs against DAPK3 (#1 and #2) and with a nonspecific control siRNA (siCtrl). On day 5 post‐transfection, HBL1 cells were stimulated with anti‐IgM for 1 h and then harvested for analysis. Expression changes were quantified using the ∆*C*_t_ method with TBP, GAPDH and PPP6C as control genes. Error bars represent the SD of three independent transfections. Significant differences calculated using two‐way ANOVA followed by Dunnett's multiple comparison test with anti‐IgM stimulated siCtrl as control. *P* values for EGR1, EGR1 +0.55 kb and EGR1 +1.0 kb = 0.0082 and 0.0009, 0.1163 and 0.2759, 0.0047 and 0.0270 for siCtrl vs. siDAPK3 #1 and siDAPK3 #2, respectively. Ns *P* > 0.05, * *P* ≤ 0.05, ** *P* ≤ 0.01, *** *P* ≤ 0.001.

Some alterations of chromatin structure during transcription favour the recruitment of the RNA processing machinery (Brown *et al.*, 2012). Since DAPKi did not repress transcription elongation, we decided to assess the role of DAPK3 in mRNA processing. For this, we performed conventional RT‐PCR looking at mature mRNAs as well as primary transcripts. Dual anti‐IgM/CD40L stimulation induced increased level of mature mRNA as well as primary transcript for both *EGR1* and *DUSP2* (Fig. 3A). Addition of ibrutinib had no effect on this transcriptional activation (Fig. 3A). DAPKi repressed mature mRNA processing and primary transcript but only if the reverse transcription was performed using an oligo‐dT but not a random primer (Fig. 3A). These data suggested that DAPK3 was participating in mRNA processing and not transcription elongation.

Having shown that DAPKi was altering mRNA processing in CLL, we decided to test if its effect was reproducible on other ibrutinib‐sensitive cells. For this, we selected a diffuse large B cell lymphoma (DLBCL) cell line, HBL1, which carries a myeloid differentiation primary response 88 (MYD88) mutation and which is sensitive to ibrutinib (Wilson *et al.*, 2015). *EGR1* and *DUSP2* expression was activated by anti‐IgM and/or CD40L stimulation in these cells, with this activation being repressed by the addition of DAPKi (Fig. S3A). Interestingly, ibrutinib did not prevent the expression of these IEGs in response to dual anti‐IgM/CD40L stimulation and could only marginally reduce the expression of *DUSP2* after anti‐IgM stimulation alone (Fig. S3A). At the same time, ibrutinib showed the strongest repressive effect in the absence of any stimulation (Fig. S3A).

Moreover, the analysis of RNA pol II recruitment in response to dual anti‐IgM/CD40L stimulation showed a similar profile compared to CLL for *EGR1* but not *DUSP2* (Figs S3B and S4). The reason why ibrutinib appeared to prevent the recruitment of the polymerase without having any effect on the expression of the studied genes was unclear. In these cells, MYD88 mutation induces chronic NF‐κB activation, which could compensate for BTK inhibition. We have shown previously that NF‐κB‐dependent activation of *TNFα* is accompanied by an accumulation of RNA pol II at the transcription end site of the gene (Thorne *et al.*, 2012), as seen for *EGR1* (Fig. S3B). Therefore, it was possible that the dynamics of RNA polymerase II recruitment to the coding region induced by NF‐κB‐dependent transcription are different when compared to other transcription factors. However, further investigations would be necessary to answer this question and are beyond the scope of this study.

In HBL1, the production of mature mRNAs and primary transcripts was altered after stimulation in presence of DAPKi (Fig. 3B). Levels of mRNAs were decreased for both IEGs and increased or unaffected for *EGR1* or *DUSP2* primary transcripts, respectively (Fig. 3B). These results confirmed that DAPK3's function in the transcription of these IEGs was the same in CLL and HBL1 cells. To confirm that DAPKi‐dependent impact on mRNA processing was not due to an off‐target effect, we performed siRNA‐mediated knockdown in HBL1 cells using two DAPK3‐specific siRNAs (Fig. 3C,D, Fig. S5). In these cells, DAPK3 knockdown was correlated with a global loss of H3T11 and H3T6 phosphorylation (Fig. 3C) and a significant loss of processed mRNA and gain of primary transcript for *EGR1* (Fig. 3D). We could not see any significant effect on *DUSP2* expression (Fig. S5), potentially due to the partial knockdown and the moderate induction of this gene in response to anti‐IgM and/or CD40L stimulation compared to *EGR*1. Nevertheless, these experiments confirm that DAPK3 control on IEG expression was post‐transcriptional.

### 3.3 DAPK inhibition blocks proliferation and both anti‐IgM‐ and CD40L‐mediated IEG expression

**Fig. 4:**
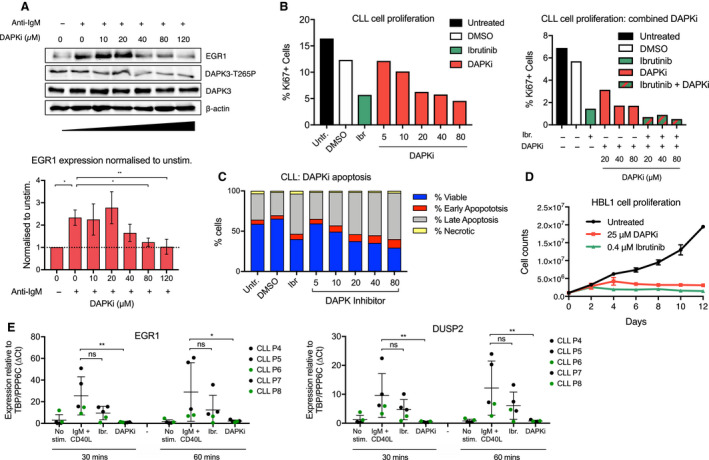
Effects of DAPK3 inhibition on CLL and lymphoid cell proliferation and cellular function. (A) Representative western blots displaying levels of EGR1 and DAPK3‐T265‐P in CLL cells pretreated with 0–120 μm DAPKi for 1 h and with or without anti‐IgM stimulation for 1 h. β‐actin was used as a loading control. Blots are normalised to unstimulated sample (unstimulated = 1.0) with image lab software (below). Error bars represent the SD of three independent experiments. Significant differences calculated using two‐way ANOVA followed by Dunnett's multiple comparison test with anti‐IgM stimulated 0 µm sample as control. *P* values = 0.0076, 0.0256 and 0.0087 for unstimulated 0 and 80 and 120 µm samples, respectively. (B) Flow cytometric analysis of CLL cells cultured on a CD40L‐expressing cell feeder layer. CLL cells were pretreated with either 1 µm ibrutinib (green), increasing concentrations (5–80 µm) of DAPKi (red), both inhibitors (green/red), or DMSO (white; negative control) for 1 h as indicated. Proliferation was assessed via the percentage of cells positive for Ki‐67. Data shown are representative of *n* = 3 CLL patients. (C) Flow cytometric analysis of CLL cells cultured on a CD40L‐expressing cell feeder layer. CLL cells were pretreated with either 1 µm ibrutinib, increasing concentrations (5–80 µm) of DAPKi or DMSO (negative control) for 1 h. Apoptosis was assessed using the Annexin V Apoptosis Detection Kit, as indicated. Proliferation was assessed via the percentage of cells positive for Ki‐67. Data shown are representative of *n* = 3 CLL patients. (D) Cell survival assay in untreated HBL1 cells compared with HBL1 cells treated with either 25 or 0.4 μm ibrutinib. Cell count was determined by trypan blue staining at 2–12 days. Error bars are representative of three independent counts. (E) qPCR data analysis of EGR1 and DUSP2 gene expression at 30–60 min post‐anti‐IgM/sCD40L stimulation in CLL cells (*n* = 5 patients). CLL cells were pretreated with 1 µm ibrutinib or 25 µm DAPKi for 1 h as indicated below the graphs. Expression changes were quantified using the ∆*C*_t_ method with TBP and PPP6C as control genes. Bars represent the grand mean of the samples for *n* = 5 patients. Significant differences calculated using two‐way ANOVA followed by Dunnett's multiple comparison test with anti‐IgM as control. EGR1 (30 m) *P* values = 0.0626 and 0.0054 for anti‐IgM vs Ibr and DAPKi, respectively. DUSP2 (30 m) *P* values = 0.1713 and 0.0086 for the same comparisons. EGR1 (60 m) *P* values = 0.1893 and 0.0245 for the same comparisons. DUSP2 (60 m) *P* values = 0.1507 and 0.0064 for the same comparisons. Ns *P* > 0.05, * *P* ≤ 0.05, ** *P* ≤ 0.01, *** *P* ≤ 0.001.

Ibrutinib blocks CLL cell proliferation *in vitro* and *in vivo*. To compare ibrutinib and DAPKi efficiency, we incubated primary CLL cells with or without these inhibitors and measured the rate of proliferation as well as the pro‐apoptotic effect of the two compounds (Fig. 4, Fig. S6). First, we pretreated CLL cells with increasing doses of DAPKi and assessed the level of EGR1 protein and phospho‐DAPK3 after anti‐IgM stimulation. DAPK3 phosphorylation at threonine 265 is essential for its full auto‐phosphorylation and enzymatic activity (Graves *et al.*, 2005). We determined that DAPKi was having an important repressive action on both proteins beyond 20 µm (Fig. 4A). Similarly, the reduction in CLL cell proliferation was comparable between ibrutinib and DAPKi for 20 µm and beyond (Fig. 4B, Fig. S6A). Moreover, combining both drugs had a synergistic effect on cell proliferation (Fig. 4B, Fig. S6A). Impact on CLL cell apoptosis correlated with the effect on proliferation, but with some variability from one experiment to another due to the small duration of the experiments performed with primary CLL (Fig. 4C, Fig. S6B). As observed for CLL cells, both ibrutinib and DAPKi blocked HBL1 cell proliferation (Fig. 4D). Finally, as suggested by the experiments performed with conventional RT‐PCR (Fig. 3A), ibrutinib did not repress *EGR1* and *DUSP2* after CLL stimulation with both anti‐IgM and CD40L compared to DAPKi, indicating that DAPKi had a broader repressive effect than ibrutinib (Fig. 4E).

## 4 Discussion

DAPK3, like other members of the DAPK family, is a pro‐apoptotic kinase with a reported tumour‐suppressor activity (Brognard *et al.*, 2011; Kawai *et al.*, 1998). DAPK3‐induced apoptosis involves both caspase‐dependent and independent pathways (Kogel *et al.*, 2001) as well as an alternative mechanism through autophagic cell death (Inbal *et al.*, 2002). However, this protein was initially identified as a chromatin‐associated enzyme phosphorylating the core histones H3, H4 and H2A (Kögel *et al.*, 1998) and more specifically H3T11 (Preuss *et al.*, 2003), suggesting that this enzyme might be involved in a wide range of functions. In line with its association with chromatin, we have shown that DAPK3 recruitment to the transcriptional machinery is enhanced in response to anti‐IgM stimulation. This kinase then targets histone H3 at threonine 6 and 11 within the body of IEGs, these PTMs being correlated with gene transcription. This mechanism does not appear to impact on transcription per se, but DAPK inhibition induces a defect in mRNA processing.

Transcription and mRNA processing are two interconnected processes, and one of the functions of the chromatin is to coordinate them (Brown *et al.*, 2012; Jimeno‐Gonzalez and Reyes, 2016). However, any potential role for the two identified histone marks in controlling mRNA processing is still unknown. In addition, it is worth considering that IEGs show rapid pre‐mRNA induction and RNA polymerase recruitment compared to secondary/delayed response genes (Bahrami and Drablos, 2016). IEGs have a distinct genomic architecture and chromatin structure to facilitate their rapid transcription characterised by bivalent promoters poised for rapid activation (Tullai *et al.*, 2007). Consequently, DAPK inhibition may be particularly effective against the pre‐mRNA processing of this class of gene. Alternatively, it cannot be excluded that DAPK3 could directly target proteins of the processing machinery.

Ibrutinib blocked DAPK3‐dependent chromatin PTMs induced by anti‐IgM stimulation by possibly preventing RNA polymerase II recruitment to the IEG promoters and gene bodies. Ibrutinib presents a breakthrough in CLL treatment, but cases of resistance which cannot be explained by genetic alterations are emerging (Ahn *et al.*, 2017; Kaur and Swami, 2017; Landau *et al.*, 2017), and recent studies indicate that alternative pro‐proliferative signalling pathways, mainly NF‐κB, were only partially blocked by this small molecule (Dadashian *et al.*, 2019; Jayappa *et al.*, 2017). Interestingly, DAPK inhibitor, like ibrutinib, prevents IEG expression and CLL and HBL1 proliferation. Off‐target effects cannot be completely ruled out at the tested inhibitor concentrations; however, ERK (IC50 > 10 μm for ERK2), the most relevant alternative target (Ten Hacken *et al.*, 2016), shows no effect on *EGR1* and *DUSP2* expression as well as on H3 phosphorylation after directly targeting MEK the enzyme activating ERK (Fig. S2) suggesting that the latter might not mediate DAPKi effect.

DAPK3 is activated downstream of BTK and PLCγ2, suggesting that it could be an attractive alternative to ibrutinib, particularly in CLL cells for which relapse is correlated with mutations of both upstream proteins. In addition, DAPKi appears to have a broader repressive function by blocking both the BCR and CD40 signalling pathways. Ibrutinib does not prevent transcription of IEGs in response to activation with CD40L as seen in our work and as described in other studies (Woyach *et al.*, 2014). Activation of CD40, a key regulator of B‐cell proliferation, augments NF‐κB activity and prolonged CLL cell survival in vitro (Furman *et al.*, 2000). Therefore, DAPK3 controlling the mRNA processing of NF‐κB target genes downstream of CD40 activation suggests it might also regulate other signalling pathways activating NF‐κB. In this respect, it has been shown that DAPK3 also regulates pro‐inflammatory genes in response to TNFα‐dependent activation (Usui *et al.*, 2012) as well as in response to interferon‐gamma (IFNγ) (Mukhopadhyay *et al.*, 2008). Remarkably for the latter, DAPK3 acts first as a repressor by activating the interferon‐gamma‐activated inhibitor of translation (GAIT), which phosphorylates the ribosomal protein L13a, a key component of this repressive complex, before GAIT‐dependent inactivation of DAPK3 will reactivate gene expression (Mukhopadhyay *et al.*, 2008). IFNγ activates a signalling cascade in which DAPK1 targets DAPK3 before DAPK3‐dependent phosphorylation of L13a (Mukhopadhyay *et al.*, 2008).

DAPK1, the first member of the death‐associated protein kinase family, can induce apoptosis in response to INFγ, TNFα and FAS pathways. Silencing of DAPK1 via hypermethylation of its promoter has been suggested to influence tumour progression and metastasis in cancers such as CLL (Raval *et al.*, 2007) and DLBCL (Kristensen *et al.*, 2014) and is a marker of poor survival. Consequently, DAPKi anti‐proliferative function in CLL and HBL1 is unlikely to be mediated by DAPK1 repression. In addition, our data suggest that, depending on the cellular context, DAPK3 could induce either proliferation and survival or cell death, the latter function being DAPK1‐dependent. By extension, DAPKi could have a repressive function of the NF‐κB pathway restricted to DAPK1‐silenced cells. Constitutive activation of NF‐κB is known to mediate cancer development as well as resistance to anti‐cancer therapies. However, targeting NF‐κB is also seen as problematic because of its key role in many physiological processes. An attractive possibility suggested by our data is that DAPK3 might repress this transcription factor only in cells with aberrant epigenetic silencing of DAPK1, therefore having a limited impact on ‘normal’ cells.

At this stage, the link between ibrutinib‐ and DAPKi‐mediated repression of IEGs and the anti‐proliferative potential of these molecules is not entirely clear. CLL cells tend to undergo rapid and spontaneous apoptosis when cultured *in vitro* (Collins *et al.*, 1989) which has been attributed to a dramatic decrease in the levels of the anti‐apoptotic protein Bcl‐2 during *in vitro* culture of CLL cells (Pepper *et al.*, 1997). Recreating the complex microenvironmental signalling network which is vital to CLL cell proliferation and survival in tissue niches *in vivo* is challenging to the extent that *ex‐vivo* stimulation of the BCR, one of the most important receptors for these cells *in vivo*, cannot induce CLL cell proliferation in culture (Schleiss *et al.*, 2019). Co‐culture of CLL cells on a CD40L‐expressing feeder layer can promote their proliferation to some extent, and some studies suggest addition of IL‐4 and IL‐21 (Schleiss *et al.*, 2019) or CpG ODN (Purroy *et al.*, 2015) can aid proliferation, but neither method allows indefinite culture or a true representation of *in vivo* conditions. We believe the discrepancies in our data may be explained somewhat by the limited culture conditions and suggest that more elaborate and developed models would be necessary to more fully recapitulate and understand CLL cell proliferation and apoptosis in response to ibrutinib and DAPK inhibition *in vitro*.

## 5 Conclusion

In conclusion, we have identified an enzyme activated downstream of the BCR signalling pathway, known as death‐associated protein kinase 3 (DAPK3). DAPK inhibition represses CLL cell proliferation *in vitro* and prevents expression of genes transcribed in response to activation of the BCR pathway. We have determined that DAPK3 is closely associated with these genes during transcription and that it participates in the processing of their mRNA. Moreover, DAPK3 inhibition appeared to have a broader effect in repressing pathways involved in cancer proliferation than ibrutinib. Our data suggest that DAPK3 inhibition could be an alternative to ibrutinib treatment in CLL, particularly in cases where resistance is associated with *BTK*/ *PLCγ2* mutations, but also more generally in cancers characterised by DAPK1‐silencing, which is a common marker of poor prognosis.

## Conflict of interest

The authors declare no conflict of interest.

## Author contributions

FT, SK and KBH conducted experiments and analysed data. FT and KBH generated figures. PFL helped with data analysis and interpretation and wrote the article. PH was involved in the initial design of the project and contributes to provide essential research material.

## Supporting information


**Fig. S1.** DAPK3/ZIPK was a candidate kinase for H3T6 and H3T11 phosphorylation.
**Fig. S2.** MEK1/2, RSK1/2 and PKCs inhibitors do not significantly inhibit IgM‐induced expression of *EGR1* and *DUSP2* in CLL cells.
**Fig. S3.** qPCR data analysis of EGR1 and DUSP2 gene expression at 30‐60 minutes post‐anti‐IgM/CD40L stimulation in HBL1 cells.
**Fig. S4.** ChIP‐qPCR data from CLL cells assessing levels of RNA polymerase II (left) and RNA polymerase II S2‐P (right) binding across the *EGR1* and *DUSP2* loci.
**Fig. S5.** qPCR data analysis of DUSP2 primary transcript in HBL1 cells transfected with siRNAs against DAPK3.
**Fig. S6.** Effects of DAPK3 inhibition on CLL cell proliferation and viability.

**Table S1.** Primers list.
