# Supplementary material for: DAPK3 participates in the mRNA processing of immediate early genes in chronic lymphocytic leukaemia
Source: Mol Oncol. 2020 May 3;14(6):1268–81. doi: 10.1002/1878-0261.12692 (PMC7266284; doi:10.1002/1878-0261.12692)
Supplement: Supplementary file 1 — Fig. S1. DAPK3/ZIPK was a candidate kinase for H3T6 and H3T11 phosphorylation. Fig. S2. MEK1/2, RSK1/2 and PKCs inhibitors do not significantly inhibit IgM‐induced expression of EGR1 and DUSP2 in CLL cells. Fig. S3. qPCR data analysis of EGR1 and DUSP2 gene expression at 30‐60 minutes post‐anti‐IgM/CD40L stimulation in HBL1 cells. Fig. S4. ChIP‐qPCR data from CLL cells assessing levels of RNA polymerase II (left) and RNA polymerase II S2‐P (right) binding across the EGR1 and DUSP2 loci. Fig. S5. qPCR data analysis of DUSP2 primary transcript in HBL1 cells transfected with siRNAs against DAPK3. Fig. S6. Effects of DAPK3 inhibition on CLL cell proliferation and viability. [file MOL2-14-1268-s001.pdf]

A

H3T6P

Kinase	Score
MEK2	4
PKCa	5.284
PKCb	4.125
PKCg	2.2
PKCd	2.435
PKCt	5.25
PKCz	3.462
RSK2	4.111
**DAPK3**	**4.056**

H3T11P

Kinase	Score
PKCa	2.873
PKCg	2.8
RSK1	3
RSK2	3.222
**DAPK3**	**6.889**

B

Table 3. Kinase Selectivity of Compound **6** As Determined in Broad Protein Kinase Panel Screening

no inhibition at 10 μM			
ABL	Erk1	MARK1	PKCζ
AKT1	FLT3	MAPKAPK2	PKD2
AKT2	FYN	MET	PKGα
AMPK	GSK3β	MSK1	PRAK
AurA	HGK	MST2	ROCK2
BTK	IGF1R	p38α	RSK1
CAMK2	INSR	PAK2	SRC
CAMK4	IRAK4	PIM2	SYK
CDK2	LCK	PKA	c-TAK1
CHK1	LYN	PKCβ2	c-Raf
CHK2			

IC_{50} > 10 μM		1 μM < IC_{50} < 10 μM	IC_{50} < 1 μM
CK1δ	FGFR1	p70S6K	DAPK1
DYRK1α	KDR		DAPK3
Erk2	SGK1		

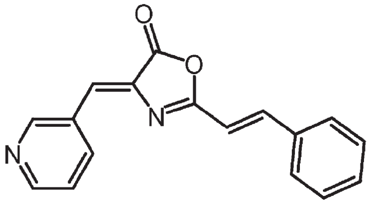

Figure S1: (A) DAPK3/ZIPK was a candidate kinase for H3T6 and H3T11 phosphorylation: *in silico* kinase specificity prediction assay software Group-based Prediction System (GPS). The GPS score is calculated as described previously (23). (B) DAPKi structure and kinase selectivity: (Okamoto et al. 2009 J. Med. Chem). DAPKi binds to active site of protein. DAPK1 IC50 = 69nM while DAPK3 IC50 = 225nM indicating moderate specificity. DAPK1 is silenced in CLL.

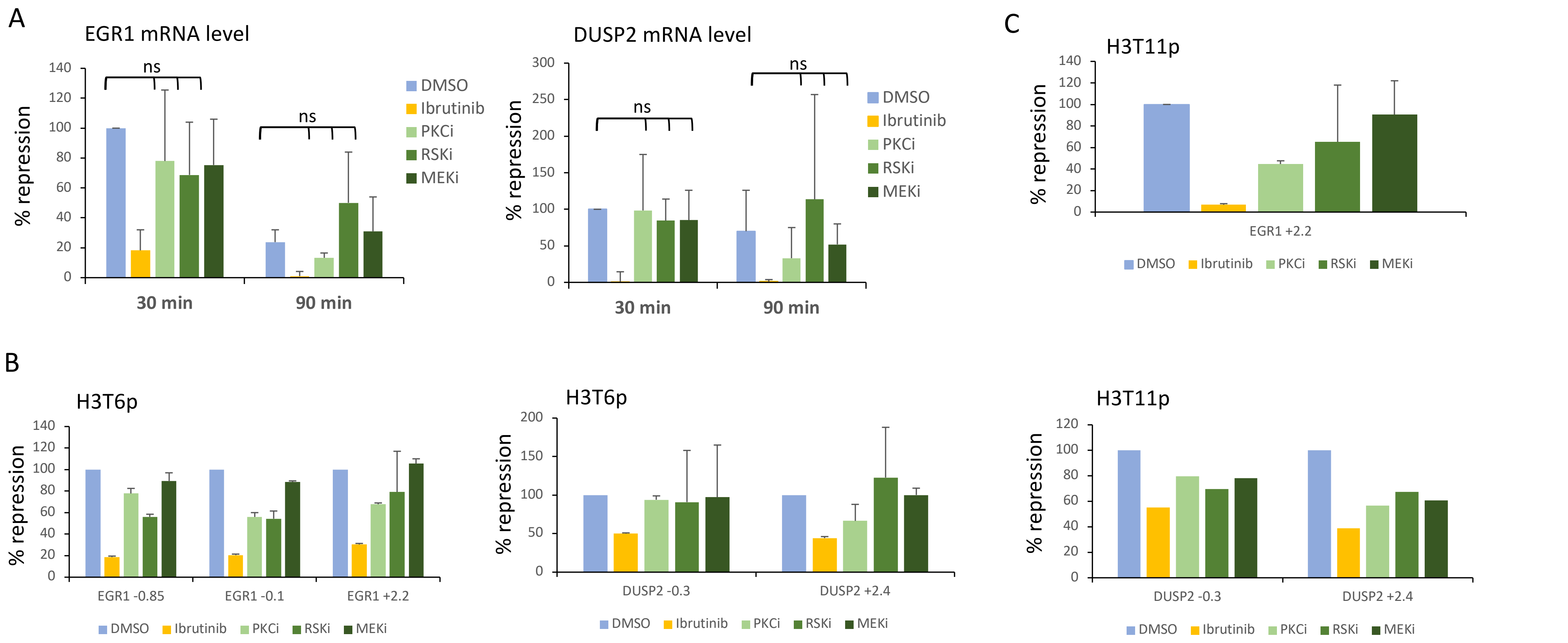

Figure S2: MEK1/2, RSK1/2 and PKCs inhibitors do not significantly inhibit IgM-induced expression of *EGR1* and *DUSP2* in CLL cells. Primary CLL cells were cultured for 48 h and then treated with DMSO, 1 µM Ibrutinib, 1 µM Gö6983 (PKCi), 10 µM RSK inhibitor II (RSKi) or 10 µM U0126 (MEKi) for 2 h, before the BCR was cross-linked. (**A**) Expression of *EGR1* and *DUSP2* was assessed and the means and SD (error bars) of at least three independent experiments from different patients are shown normalised to *TBP* and to the induction of *EGR1* or *DUSP2* mRNA expression in DMSO-treated cells after 30 min (ns > 0.05 in Student's t test). (**B and C**) ChIP was performed before and after 30 min anti-IgM stimulation using primers along the *EGR1* and *DUSP2* genes (numbers indicate the positions of amplicons versus the transcription start site) and antibodies against H3 and (**B**) H3T6p or (**C**) H3T11p. ChIP results after 30 min anti-IgM stimulation are plotted. Results of one representative patient are shown normalised to H3 and the average of the CTCF1 and CTCF3 control regions and, then, versus, unstimulated DMSO-treated cells. Error bars represent the standard deviation (SD) for three independent replicates.

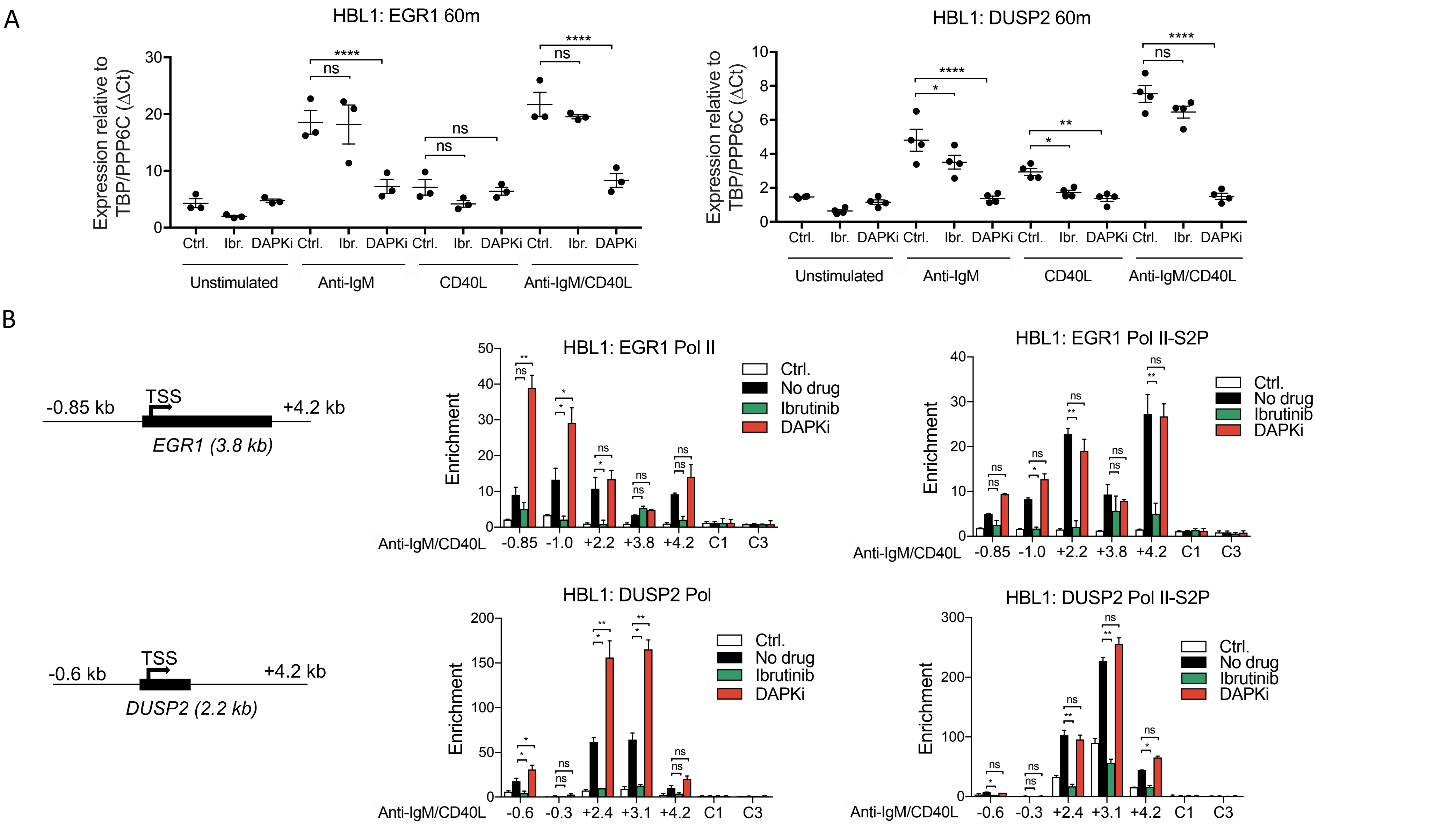
A
HBL1: EGR1 60m
Expression relative to TBP/PPP6C (ΔCt)
30
20
10
0
ns
****
ns
ns
ns
****
Ctrl.
Ibr.
DAPKi
Unstimulated
Anti-IgM
CD40L
Anti-IgM/CD40L
HBL1: DUSP2 60m
10
8
6
4
2
*
**
****
B
-0.85 kb
TSS
+4.2 kb
EGR1 (3.8 kb)
-0.6 kb
TSS
+4.2 kb
DUSP2 (2.2 kb)
HBL1: EGR1 Pol II
Enrichment
50
40
Ctrl.
No drug
Ibrutinib
DAPKi
Anti-IgM/CD40L
-0.85
-1.0
+2.2
+3.8
+4.2
C1
C3
HBL1: EGR1 Pol II-S2P
HBL1: DUSP2 Pol
200
150
100
50
-0.6
-0.3
+2.4
+3.1
HBL1: DUSP2 Pol II-S2P
300

Figure S3: (A) qPCR data analysis of EGR1 and DUSP2 gene expression at 30-60 minutes post anti-IgM/CD40L stimulation in HBL1 cells. Cells were treated with ibrutinib or DAPKi as indicated below the graphs. Expression changes were quantified using the ∆Ct method with TBP and PPP6C as control genes. Error bars represent the SEM of 3 independent experiments. Significant differences calculated using two-way ANOVA followed by Dunnett's multiple comparison test with ctrl as the control. EGR1 P values = 0.9960, 0.0058, 0.0069, 0.5620, 0.2339 & 0.0001 for ctrl vs ibrutinib and DAPKi with anti-IgM, CD40L and anti-IgM/CD40L stimulation, respectively. DUSP2 P values = 0.0599, 0.0002, 0.0004, 0.0001, 0.0383 & 0.0001 for the same comparisons. **(B)** ChIP-qPCR data from HBL1 cells assessing levels of RNA polymerase II (top), RNA polymerase II S2-P (bottom) binding across the EGR1 and DUSP2 loci at 45 minutes post stimulation with both anti-IgM and sCD40L. HBL1 cells were pre-treated with either 1 µM ibrutinib (green) or 25 µM DAPKi (red) for 1 hour as indicated. The values on the x axis refer to specific gene regions relative to the transcription start site (TSS) in kilobases (kb) as indicated on the gene schematics below (not to scale). CTCF1/3 (C1/C3) were used as negative control regions which are not indicated on the gene schematics. Error bars correspond to variations in the ChIP-qPCR repeats. The stars represent the significance, calculated via two-way ANOVA followed by Dunnett's multiple comparison test, to compare anti-IgM with Ibr and DAPKi, between 3 replicate experiments in HBL1 cells. Pol II P values = 0.0035 (EGR1 -0.85), 0.0338 & 0.0395 (EGR1 -1.0), 0.0056 (EGR1 +2.2), 0.0483 & 0.0414 (DUSP2 -0.6), 0.0274 & 0.0043 (DUSP2 +2.4) and 0.0381 & 0.0075 (DUSP2 +3.1). Pol II S2-P P values = 0.0226 (EGR1 -1.0), 0.0031 (EGR1 +2.2), 0.0028 (EGR1 +4.2), 0.0478 (DUSP2 -0.6), 0.0091 (DUSP2 +2.4), 0.0065 (DUSP2 +3.1) and 0.0259 (DUSP2 +4.2).

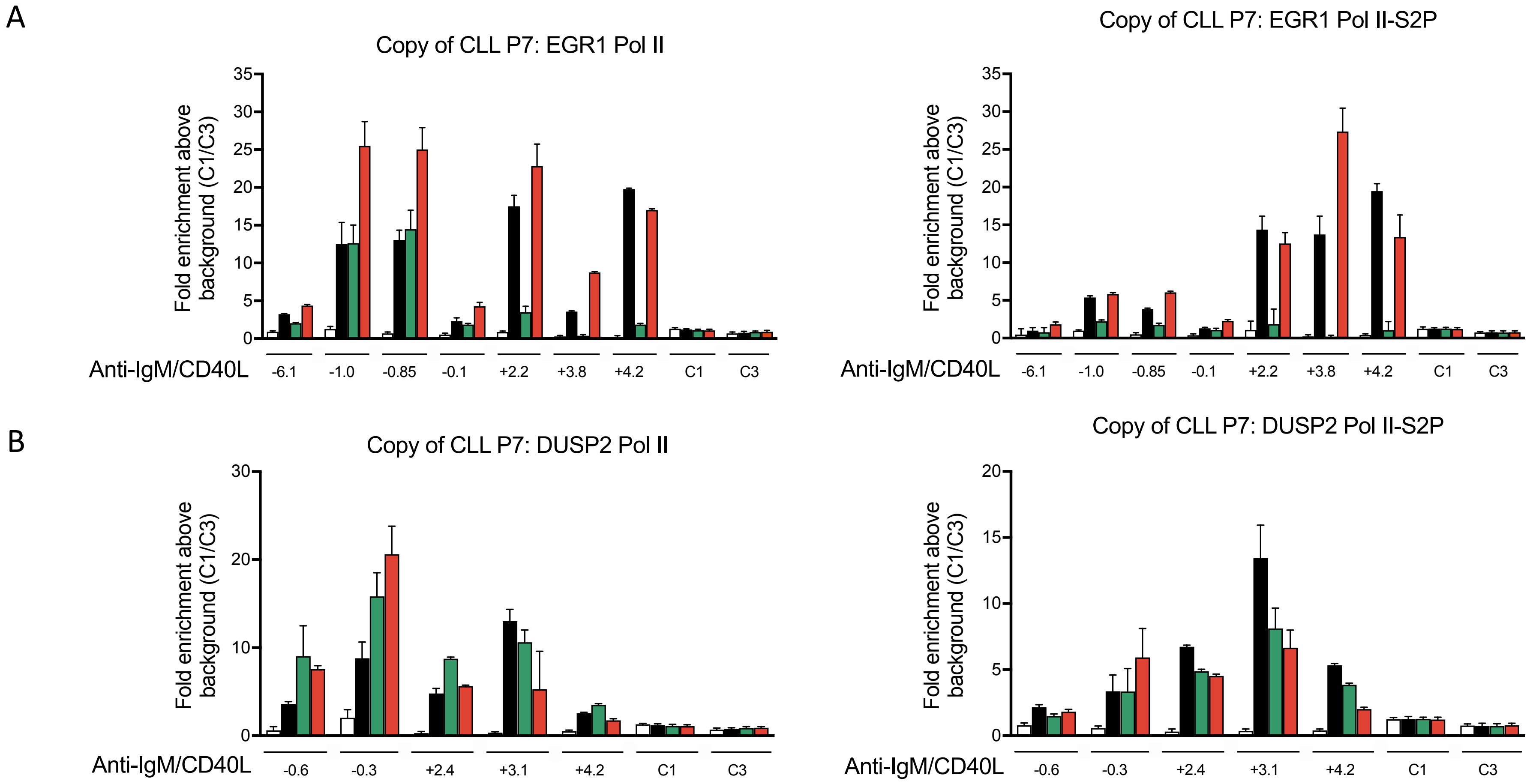

Figure S4: ChIP-qPCR data from CLL cells assessing levels of RNA polymerase II (left) and RNA polymerase II S2-P (right) binding across the **(A)** *EGR1* and **(B)** *DUSP2* loci at 30 minutes post stimulation with combined anti-IgM/CD40L. CLL cells were pre-treated with either 1 μM ibrutinib (green) or 25 μM DAPKi (red) for 1 hour as indicated. The values on the x axis refer to specific gene regions relative to the transcription start site (TSS) in kilobases (kb) as indicated on the gene schematics below (not to scale). CTCF1/3 (C1/C3) were used as negative control regions which are not indicated on the gene schematics. Error bars correspond to variations in the ChIP-qPCR repeats performed in triplicates.

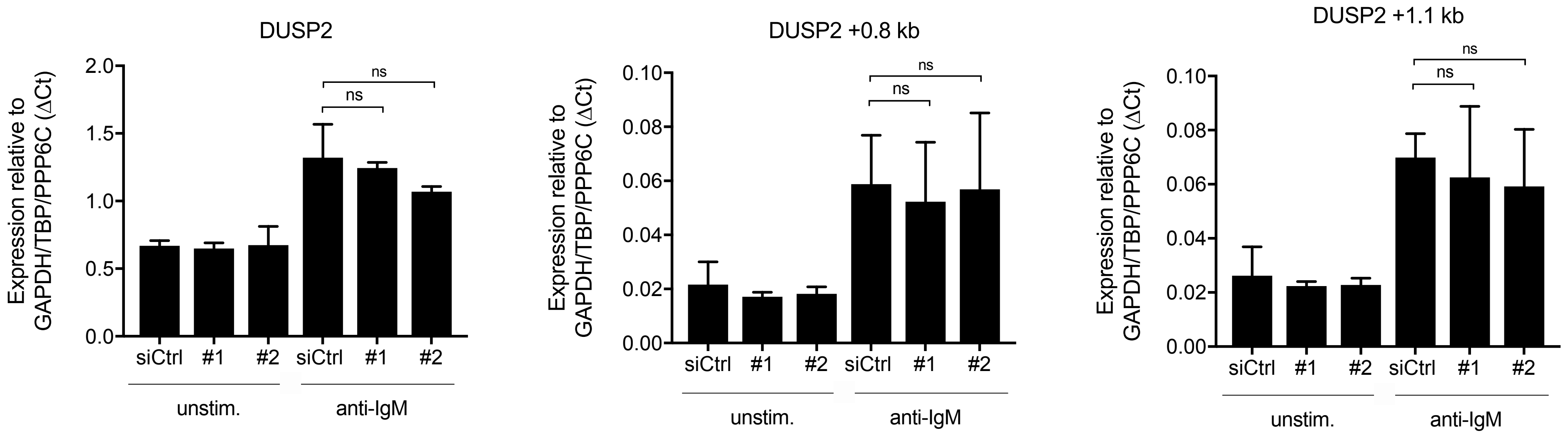

Figure S5: qPCR data analysis of EGR1 primary transcript (E and F) and processed mRNA (D) at 60 minutes post-IgM stimulation in HBL1 cells transfected with siRNAs against ZIPK (#1 and #2) and with a non-specific control siRNA (siCtrl). Expression changes were quantified using the ∆Ct method with TBP, GAPDH and PPP6C as control genes. Error bars representative of 2 independent transfections. Error bars represent the SD of 3 independent transfections. Significant differences calculated using two-way ANOVA followed by Dunnett's multiple comparison test with anti-IgM stimulated siCtrl as control. P values for DUSP2 = 0.8242 and 0.0995, DUSP2 +0.8 kb = 0.9550 and 0.9997 and DUSP2 +1.1 kb = 0.9070 and 0.7358 for siCtrl vs. siZIPK #1 & siZIPK #2 respectively.

A B

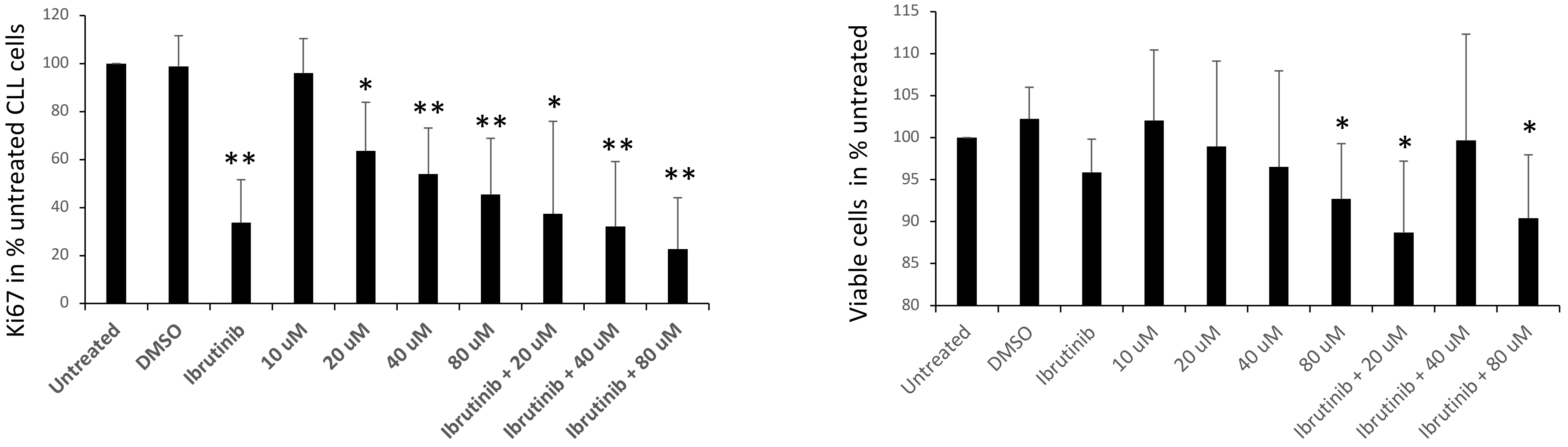

Figure S6: Effects of DAPK3 inhibition on CLL cell proliferation and viability. (**A-B**) Flow cytometric analysis of CLL cells cultured on a CD40L-expressing cell feeder layer. CLL cells were pre-treated with either ibrutinib, increasing concentrations (10-80 µM) of DAPKi, both inhibitors, or DMSO for 1 hour as indicated. (**A**) Proliferation was assessed via the percentage of cells positive for Ki-67. (**B**) Apoptosis was assessed using the Annexin V Apoptosis Detection Kit. **(A-B)** Error bars represent the standard deviation calculated from 4 independent experiments (** < 0.01, * < 0.05, ns > 0.05 in Student’s t test).
