# Supplementary material for: DAPK3 participates in the mRNA processing of immediate early genes in chronic lymphocytic leukaemia
Source: Mol Oncol. 2020 May 3;14(6):1268–81. doi: 10.1002/1878-0261.12692 (PMC7266284; doi:10.1002/1878-0261.12692)
Supplement: Supplementary file 2 — Table S1. Primers list. [file MOL2-14-1268-s002.pdf]

CTCF1 F	GGCCCAGGACTCCACGTTCAGA	ChIP
CTCF1 R	GCCCTCTGGTGTTTGGCAGCAA	ChIP
CTCF3 F	CACCCAGCAGAGGGCCCAGATA	ChIP
CTCF3 R	CCCTTCGCCTTCTCTCCAGCCA	ChIP
DUSP2 -0.3 F	CTAAAGGGGGACCTGCATCTG	ChIP
DUSP2 -0.3 R	GACTAAGGGAAGGGAAGGTGTT	ChIP
DUSP2 -0.6 F	AGGTCTTGAAATCCCCGCAG	ChIP
DUSP2 -0.6 R	CCAGGCCCCAGAGAGAAGTA	ChIP
DUSP2 -11.0 F	CCTTGACTCCCTGCTGAAGG	ChIP
DUSP2 -11.0 R	CCCATCACAGCCCAAGTCTT	ChIP
DUSP2 +0.5 F	GCTGGTTTTGTCCCCTGTTG	qPCR/RT-PCR
DUSP2 +0.5 R	CTGATCGTGCTCTTCCCCTC	qPCR/RT-PCR
DUSP2 +0.8 F	CCAGTCCTCCTGACCTGAGA	qPCR/RT-PCR
DUSP2 +0.8 R	GTGCCTGTATGGGTCTGTGT	qPCR/RT-PCR
DUSP2 +1.1 F	GGTGCCCCCTTACCAATGAA	qPCR/RT-PCR
DUSP2 +1.1 R	GGGCCTTTTCCGCTACAAGA	qPCR/RT-PCR
DUSP2 +2.4 F	GGAAGTGATGGGTGTGTCATGT	ChIP
DUSP2 +2.4 R	AAATAATTTTCCAGCGCCAGCA	ChIP
DUSP2 +3.1 F	CATTTGGAGTGTCATCCAGCCA	ChIP
DUSP2 +3.1 R	ACAGACCACGGACAGATCCTTA	ChIP
DUSP2 +4.2 F	AGGAGTGAGGTTGATCTGATGT	ChIP
DUSP2 +4.2 R	CCAGCCGTTATGAGTGTCCAT	ChIP
DUSP2 F	TACTTCCTGCGAGGAGGCTT	qPCR/RT-PCR
DUSP2 R	GGCTGGTTTTGTCCCCTGTT	qPCR/RT-PCR
EGR1 -0.1 F	GCCATATTAGGGCTTCCTGCTT	ChIP
EGR1 -0.1 R	GATCCGCCTCTATTTGAAGGGT	ChIP
EGR1 -0.4 F	TCTGGGAGGAGGGAAGAAGG	ChIP
EGR1 -0.4 R	TTCGGGGAAGCCTAGAGC	ChIP
EGR1 -0.85 F	TACAGTGTCCCAAGAACCAAGT	ChIP
EGR1 -0.85 R	CGATCTATGGCACGGTGTCTTT	ChIP
EGR1 -1.0 F	CCCTCCACCTGGACTGGATA	ChIP
EGR1 -1.0 R	CCGAAGTGGGGAAGCTGATT	ChIP
EGR1 -6.1 F	CCTGGGGTAGGAGCAGAACT	ChIP
EGR1 -6.1 R	TGTCACACTTTCCGACTGACTT	ChIP
EGR1 +0.55 F	CAGCACCTTCAACCCTCAGG	qPCR/RT-PCR
EGR1 +0.55 R	ATGGTGGCGAAAGGGTTCC	qPCR/RT-PCR
EGR1 +1.0 F	TCCGTATTTGCGTCAGCTGT	qPCR/RT-PCR
EGR1 +1.0 R	GCTACCATTGACTCCCGAGG	qPCR/RT-PCR
EGR1 +2.2 F	CTGCGACATCTGTGGAAGAAAG	ChIP
EGR1 +2.2 F	GCCGCAAGTGGATCTTGGTAT	ChIP
EGR1 +3.8 F	GCTGCGATTGGGTATGTGTTTC	ChIP
EGR1 +3.8 R	AGAAAACCTCACAGCCCTCAAT	ChIP
EGR1 +4.2 F	GGGCCTGTTCCTCTTCAGTC	ChIP
EGR1 +4.2 F	CACACATGTCCCAGCCCTAA	ChIP
EGR1 F	ACAAAAGTGTTGTGGCCTCTTC	qPCR/RT-PCR
EGR1 R	GTGGCCGGGGATGGATAAGA	qPCR/RT-PCR
EGR1 TSS F	GCCATATTAGGGCTTCCTGCTT	ChIP
EGR1 TSS R	GATCCGCCTCTATTTGAAGGGT	ChIP

GAPDH F	ACAGTCAGCCGCATCTTCTT	qPCR
GAPDH R	ACGACCAAATCCGTTGACTC	qPCR
PPP6C F	TGGAAATGCTAATGCCTGGAGA	qPCR
PPP6C R	TTCCTGATTCCGTTCGATGGTT	qPCR
TBP F	TGCTCACCCACCAACAATTTAG	qPCR
TBP R	TCTGGGTTTGATCATTCTGTAGATTAA	qPCR
